# Synchronizing and desynchronizing effects of nonlinear delayed feedback deep brain stimulation in Parkinson’s disease

**DOI:** 10.1186/1471-2202-13-S1-P53

**Published:** 2012-07-16

**Authors:** Andrey A Dovzhenok, Choongseok Park, Robert M Worth, Leonid L Rubchinsky

**Affiliations:** 1 Department of Mathematical Sciences and Center for Mathematical Biosciences, Indiana University Purdue University Indianapolis, Indianapolis, IN 46202, USA; 2 Department of Neurosurgery, Indiana University School of Medicine, Indianapolis, IN 46202, USA; 3 Stark Neurosciences Research Institute, Indiana University School of Medicine, Indianapolis, IN 46202, USA

## 

Hypokinetic symptoms of Parkinson’s disease have been related to the excessive beta-band oscillations and synchrony in basal ganglia and other structures; suppression of these synchronized oscillations is believed to suppress the motor symptoms of Parkinson’s disease [1]. This leads to a strong interest in a possibility of desynchronizing these pathological oscillations through electrical stimulation of affected subcortical areas. Deep brain stimulation (DBS) is an accepted clinical practice, however, no feedback-based DBS algorithm has being implemented, which would desynchronize neural activity by delivering small currents. One method received a lot of attention recently and has appeared to be very promising: nonlinear delayed feedback control. Numerical simulation showed that a delayed feedback through the mean field (being a proxy for easy-to-record local field potentials, LFP) may cancel the effect of coupling and desynchronize ensembles of coupled oscillators (see, for example, [2]). When the dynamics became desynchronized, the stimulation currents needed to support it were very small (vanishing in the large number of neurons limit). Subsequent studies provided further computational evidence for the ability of nonlinear delayed feedback to destabilize synchronized state and thus to desynchronize synchronous dynamics.

However, the dynamics of the neural activity in Parkinson’s disease exhibits complex intermittent synchronous patterns [3], far from the idealized synchronous dynamics used to study the delayed feedback stimulation. To investigate the action of nonlinear delayed feedback stimulation on the parkinsonian synchrony we employ the computational model of the basal ganglia networks [4], which successfully reproduces experimentally recorded neural activity, as ensured by the matching the temporal patterning of the synchronous dynamics [5]. The model [4] is based on the membrane properties of the basal ganglia cells and is can reproduce not only the average synchrony levels, but also the temporal patterns of the synchronous dynamics, recorded in experiment.

We study the effect of delayed feedback in numerical experiment. When the parameters of our model are such that the dynamics is strongly synchronous, the feedback, in line with earlier studies, exerts desynchronizing action. However, this strongly synchronous dynamics is not physiologically realistic. When the model is tuned to reproduce highly variable temporal patterns observed experimentally, the same delayed feedback tends to increase the synchrony in the system. “Desynchronizing” delayed feedback acting on realistic partially synchronous dynamics boosts rather than suppresses synchronization. The study also indicates that in general, delayed feedback stimulation may not necessarily exhibit desynchronization effect, when acting on a realistic partially synchronous dynamics (the kind of dynamics expected in neuroengineering applications), and sets an example of how to estimate the stimulation effect.
